# Global prognostic impact of driver genetic alterations in patients with lung adenocarcinoma: a real-life study

**DOI:** 10.1186/s12890-021-01803-0

**Published:** 2022-01-10

**Authors:** Panagiotis Paliogiannis, Maria Colombino, Maria Cristina Sini, Antonella Manca, Milena Casula, Grazia Palomba, Marina Pisano, Valentina Doneddu, Angelo Zinellu, Davide Santeufemia, Sardinian Lung Cancer (SLC) Study Group, Pietro Pirina, Alessandro Giuseppe Fois, Carlo Putzu, Giorgio Astara, Mario Scartozzi, Anna Maria Carta, Giuseppe Porcu, Gianfranco Bardino, Claudio Sini, Francesca Capelli, Maria Giuseppina Sarobba, Giovanni Sotgiu, Antonio Cossu, Giuseppe Palmieri

**Affiliations:** 1 Department of Medical, Surgical and Experimental Sciences, University of Sassari, 07100 Sassari, Italy; 2 Unit of Cancer Genetics, Institute of Biomolecular Chemistry (ICB), National Research Council (CNR), 07100 Sassari, Italy; 3 Unit of Cancer Genetics, Institute of Genetic and Biomedical Research (IRGB), National Research Council (CNR), Traversa La Crucca 3, 07100 Sassari, Italy; 4 Department of Biomedical Sciences, University of Sassari, 07100 Sassari, Italy; 5 Medical Oncology, Civil Hospital, 07041 Alghero (SS), Italy

**Keywords:** Lung adenocarcinoma, Mutation analysis, *EGFR*, *KRAS*, *BRAF*, *ALK* and *MET* rearrangements, Prognosis

## Abstract

**Background:**

Advanced lung adenocarcinoma (LAC) is one of the most lethal malignancies worldwide. The aim of this study was to evaluate the global survival in a real-life cohort of patients with LAC harboring driver genetic alterations.

**Methods:**

A series of 1282 consecutive Sardinian LAC patients who underwent genetic testing from January 2011 through July 2016 was collected. Molecular tests were based on the clinical needs of each single case (*EGFR*-exon18/19/21, *ALK*, and, more recently, *BRAF*-exon15), and the availability of tissue (*KRAS*, *MET*, and presence of low-frequency EGFR-T790M mutated alleles at baseline).

**Results:**

The mean follow-up time of the patients was 46 months. *EGFR*, *KRAS*, and *BRAF* mutations were detected in 13.7%, 21.3%, and 3% of tested cases, respectively; *ALK* rearrangements and *MET* amplifications were found respectively in 4.7% and 2% of tested cases. As expected, cases with mutations in exons 18–21 of *EGFR*, sensitizing to anti-EGFR tyrosine kinase inhibitors (TKIs) agents, had a significantly longer survival in comparison to those without (*p* < 0.0001); conversely, *KRAS* mutations were associated with a significantly lower survival (*p* = 0.0058). Among LAC patients with additional tissue section available for next-generation sequencing (NGS)-based analysis, 26/193 (13.5%) patients found positive for even low-rate EGFR-T790M mutated alleles at baseline were associated with a highly significant lower survival in comparison to those without (8.7 vs. 47.4 months, *p* < 0.0001).

**Conclusions:**

In addition to its predictive value for addressing targeted therapy approaches, the assessment of as more inclusive mutation analysis at baseline may provide clues about factors significantly impacting on global survival in advanced LAC patients.

## Introduction

Lung cancer is currently one of the most incident and lethal malignancies; in accordance with data from the Global Cancer Observatory, in 2020 were estimated more than 2.2 million new cases and approximately 1.8 million deaths worldwide, and a continuously increasing trend for both the incidence and mortality rates is expected for the next 20 years [1]. The narrowness in the gap between incidence and mortality rates, witnesses the difficulties in the clinical management of patients with lung cancer, especially those diagnosed with advanced stage disease, and the persistence of high mortality rates in this subset of patients. It is currently estimated that less than 21% of lung cancer patients are alive after five years from diagnosis [2], and this depends on several factors, such as the silent clinical course of the disease that leads to a late diagnosis in most cases, advanced age, impaired lung function related with tobacco smoking, cardiovascular and other comorbidities, histological subtype of the disease, and others [3, 4]. Significant improvements in lung cancer survival have been obtained in the last decade with the introduction of two novel therapeutic approaches for patients affected by non-small cell lung cancer (NSCLC), a group of histological subtypes that includes approximately 85% of lung cancers: immunotherapy and gene targeted therapy.

Immunotherapy with immune check point inhibitors (ICIs) is currently available for all programmed death 1 (PD1)/programmed death ligand 1 (PDL1) positive NSCLC subtypes, while targeted therapies are limited to patients with lung adenocarcinoma harboring specific genetic alterations like *EGFR*, *BRAF*, and *MET* mutations, as well as *ALK*, *ROS1*, and *RET* rearrangements or *NRTK1/2/3* gene fusions [5].

Tyrosine Kinase Inhibitors (TKIs) against mutations of the *EGFR* gene were the first to introduce survival benefits in patients with lung adenocarcinoma, opening a new era in this setting. Several clinical trials reported improved outcomes with first (i.e. gefitinib, erlotinib) and second (i.e. afatinib, dacomitinib) generation anti-EGFR TKIs, which however were time-limited because of the occurrence of resistance against these drugs, especially in patients with tumors harboring specific mutations, like the T790M mutation in exon 20 of the *EGFR* gene [6]. Currently a third generation anti-EGFR TKI, which overcomes most (but not all) the known resistances, is available (Osimertinib). Survival improvements have been mainly documented in clinical trials designed to investigate specific drugs or treatment combinations, but less is known from real-life studies investigating the prognostic impact in daily practice-selected patients. In addition, mutations in other genes like *KRAS*, *BRAF* or *HER2* have been reported to be negative prognostic biomarkers in patients with lung adenocarcinoma, making the prognostic landscape more complex [7]. In the present study we investigated the global survival rates in a cohort of Sardinian patients with lung adenocarcinoma harboring driver genetic alterations in the *EGFR*, *KRAS*, *BRAF*, *ALK* and *MET* genes with the aim to examine their real-life impact on survival, and potential correlations with several demographic, life-habit and clinical factors.

## Materials and methods

### Patients

A series of consecutive Sardinian patients with a histologically proven diagnosis of locally advanced or metastatic lung adenocarcinoma who underwent genetic molecular testing were retrospectively enrolled from January 2011 through July 2016. The demographic and clinical data at the time of diagnosis were retrieved from medical records and pathology reports. Survival data were retrieved from the Cancer Registry of the Province of Sassari, which makes part of a wider web of tumor registries coordinated by the Italian Association for Tumor Registries (Associazione Italiana Registri Tumori, AIRTUM) [4]. Sardinian origin was ascertained through verification of the place of birth for all patients. All patients were informed about the aims of this study and, before the tissue sample was collected, provided written informed consent. The study was performed in accordance with the principles of the Declaration of Helsinki and was approved by the Committee for the Ethics of the Research and Bioethics of the National Research Council (CNR).

### Molecular testing

For molecular testing, formalin-fixed paraffin-embedded (FFPE) tissue sections containing at least 80% of malignant cells from each tumor were obtained; in cases with lower neoplastic cell content, tissue sections underwent tumor macro-dissection using a single edge razor blade and a marked haematoxylin/eosin slide as a guide to remove unwanted tissue parts. All samples were processed at the Unit of Cancer Genetics of the National Research Council in Sassari (Italy), which performed routine molecular testing for all the Sardinian hospitals in the period of the study.

The molecular tests to perform were based on the clinical needs of each single case (mutations in exons 18, 19, and 21 of *EGFR* as firstly-required test; immediately afterwards, *ALK* rearrangements), and the availability of tissue to submit in further analysis of three genes not used in clinical practice for targeted therapies at the time of the study (mutations in all coding exons of *KRAS* and exon 15 of *BRAF*; *MET* rearrangements), but with active involvement in the pathogenesis of lung cancer. Overall, all patients who were screened for other driver mutations in addition to the *EGFR* ones underwent genetic analysis for all the remaining genes (*KRAS*, *ALK*, *BRAF*, *MET*) independently on the positive or negative result by the *EGFR* mutation testing.

In our series, *EGFR*-mutated patients were treated with first (Gefitinib, Erlotinib) or second generation (Afatinib) anti-EGFR TKI, whose duration of efficacy varied due to the acquisition of drug resistance, mainly based on occurrence of the T790M mutation in exon 20 of the *EGFR* gene (data not shown; Casula et al., manuscript in preparation).

For mutation analysis genomic DNA was isolated from tissue sections using a standard protocol, and DNA quality was assessed for each specimen, as previously described [8]. Briefly, paraffin was removed from formalin-fixed paraffin-embedded (FFPE) samples by treatment with Bio-Clear (Bio-Optica, Milan, Italy), and DNA was purified using the GeneRead DNA FFPE Kit, (Qiagen Inc., Valencia, CA, USA) following the manufacturer’s instructions. Yields of purified DNA were assessed by the Qubit dsDNA High-Sensitivity Assay Kit on the Qubit 2.0 Fluorometer (Life Thermofisher, Waltham, MA USA). Mutation analysis was conducted in the coding sequence of the following genes: *EGFR* (exons 18, 19, and 21), *KRAS* (exons 2, 3, and 4), and *BRAF* (exon 15). Quantitative measurements of mutations were based on pyrosequencing performed on a Pyro- Mark Q24 system (Qiagen Inc., USA) with a detection limit of 5–7%, following the manufacturer’s instructions [9]

Next-generation sequencing (NGS) was performed in 193 FFPE tissues using Ion S5-GeneStudio System and carried out by Ion Oncomine™ Focus Gene Assay which provides multiplexed target selection of 35 hotspot genes implicated in cancer research. Starting DNA and libraries were accurately quantified using a fluorescence-based quantification method, such as Qubit dsDNA HS. Result filtering, annotation, and data analysis workflow was performed by automated data transfer, from the Ion Torrent™ Server to the Ion Reporter Server for variant analysis. Coverage of > 300 reads and frequency of mutated alleles > 3% for gene amplicon, in order to get a total amount of at least 10 mutated alleles for each candidate amplicon, were usually adopted for mutation selection criteria at somatic level. To even verify the existence at baseline of tumor subclones carrying the EGFR-T790M variant, we arbitrarily decided to go below the above-mentioned threshold value in order to identify up to few mutated alleles—ranging from 3 (0.15%) to 28 (1.47%) EGFR-T790M variants with a coverage of about 1900 reads—using the Integrative Genomics Viewer (IGV) tool.

Fluorescence in situ hybridization (FISH) for *MET*, was carried out in interphase tumor cells using the specific CTB.13 N12 BAC probe (at the 7q31.2 locus) and the control centromere, labeled with Spectrum-Orange and Spectrum-Green (Vysis, Downer’s Grove, IL, USA), respectively. For *ALK*, we used the ALK Break Apart FISH Probe Kit (Vysis, USA), as the first methodology deployed widely according to the recommendations by the National Comprehensive Cancer Network/NCCN guidelines (Version 3.2011). For *ALK*, the presence of rearrangement was defined when ≥ 15% of cells were positive for FISH signals according to the indications provided for the ALK Break Apart FISH Probe Kit (Vysis, USA). Amplification of the *MET* gene was defined by the presence of at least one of the following: (a) candidate gene to control centromere ratio ≥ 2, according to the main criterion provided for assessing *EGFR* gene copy number in NSCLC; and/ or (b) presence of at least a tetrasomic signal (≥ 2.0 gene copies per control centromere) in more than 15% of cells.

### Statistical analysis

Descriptive analysis for qualitative and quantitative variables was conducted using proportions and the mean ± standard deviation (SD) or median and interquartile range (IQR), respectively. Statistical differences between groups were compared using unpaired Student’s *t*-test, Mann–Whitney rank sum test, chi-square test or Fisher’s exact test as appropriate. Survival was investigated with Kaplan–Meier and Cox regression analyses. *p* ≤ 0.05 was considered statistically significant. Data were analyzed using MedCalc for MS Windows, version 15.4 64 bit (MedCalc Software, Ostend, Belgium).

## Results

A total of 1440 patients with lung adenocarcinoma who underwent genetic test to establish the presence of targeted genetic alterations in the period under investigation were identified. Among them, 158 were excluded because of lack of detailed clinical and/or follow-up data. Finally, 1282 patients were enrolled. Table 1 summarizes the global demographic, clinical and mutational data of the patients enrolled.

**Table 1:** Demographic, clinical and mutational data of the patients included in the study

Total cases	1282
Age, median (IQR)	67 (60–73)
≤ 50, n (%)	100 (8.5)
> 50, n (%)	1182 (91.5)
Males, n (%)	848 (66)
Never smokers/ data available, n (%)	140/780 (17.9)
*EGFR* mutated/ analyzed (%)	176/1282 (13.7)
Exon 18, n (%)	11 (6.2)
Exon 19, n (%)	89 (50)
Exon 21, n (%)	78 (43.8)
*KRAS* mutated/analyzed (%)	201/944 (21.3)
Exon 2, n (%)	171 (85.1)
G12C	68 (39.8)
G12V	38 (22.2)
G12D	34 (19.9)
Other G12	21 (12.3)
G13D	10 (5.8)
Exon 3, n (%)	30 (14.9)
Q61H	15 (50)
Q61L	15 (50)
*BRAF* V600E mutated/analyzed (%)	28/944 (3)
*ALK* rearranged/analyzed (%)	41/880 (4.7)
*MET* amplified/analyzed (%)	14/692 (2)
Concomitant genetic alterations	
* EGFR* + *MET*, n (%)	2 (0.3)
* ALK* + *MET*, n (%)	2 (0.3)
Follow-up time, mean (± SD), months	46.1 (± 27.4)
Survival, mean (± SD), months	19.9 (± 22.4)

*EGFR* mutation analysis was performed in all the 1282 cases, as it was the first to be introduced in clinical practice, while *KRAS* and *BRAF* testing was carried out in 944 cases with tissues available to analyze. As stated in Methods, all patients with available tumor tissue sample were screened for mutations in the entire series of additional candidate genes (*KRAS*, *ALK*, *BRAF*, *MET*) regardless of the positive or negative result of the *EGFR* mutation testing.

The study of *ALK* rearrangements started with the adoption of the test in clinical practice in 2012 and involved 880 patients, and since then also *MET* amplifications were searched in 692 cases with available samples. Eight hundred forty-eight (66%) of the patients enrolled were males, and the median age was 67 (IQR 60–73) years (Table 1). *EGFR*, *KRAS*, and *BRAF* mutations were detected in 176 (13.7%), 201 (21.3%), and 28 (3%) of tested cases, respectively. *ALK* rearrangements and *MET* amplifications were found respectively in 41 (4.7%) and 14 (2%) of tested cases. In two cases, *EGFR* simultaneous mutations in different exons were observed; only rare concomitant genetic alterations in different genes were observed (Table 1). The exons affected in each single mutated gene are summarized in Table 1. Further details on the specific subtypes of the genetic alterations found and their correlation with demographic, pathologic and smoking exposure data have been previously published in a larger series partially containing the current cases [10].

*EGFR* mutations were significantly more common in females and never smokers, as opposed to *KRAS* mutations which were significantly more frequent in males and ever smokers (Table 2). *ALK* rearrangements were more common in younger patients, with the difference being close to statistical significance. *BRAF* mutations were more common in males and smokers, but these differences did not reach statistical significance, while *MET* amplifications were significantly more frequent in females than in males (Table 2).

**Table 2:** Genetic alterations in accordance with the gender, smoking habit, and age of the patients tested

Males	Females	*p*	Never smokers	Ever smokers	*p*	Age ≤ 50	Age > 50	*p*
***EGFR* (n = 1282)**								
62/848 (7.3%)	114/434 (26.3)	**< 0.0001**	62/140 (44.3%)	114/641 (17.8%)	**< 0.0001**	18/100 (18%)	158/1182 (13.4%)	0.2537
***KRAS* (n = 944)**								
156/619 (25.2%)	45/325 (13.8%)	**0.0001**	14/133 (10.5%)	187/585 (32%)	**< 0.0001**	18/83 (21.7%)	183/861 (21.2%)	0.9613
***BRAF* (n = 944)**								
20/619 (3.2%)	8/325 (2.5%)	0.6453	2/133 (1.5%)	26/585 (4.4%)	0.1825	2/83 (2.4%)	26/861 (3%)	0.9794
***ALK* (n = 880)**								
26/592 (4.4%)	15/288 (5.2%)	0.7123	5/82 (6.1%)	36/398 (9%)	0.5140	6/57 (10.5%)	35/823 (4.2%)	0.0645
***MET* (n = 692)**								
5/471 (1%)	9/221 (4.1%)	**0.0247**	2/75 (2.7%)	12/345 (3.5%)	1.000	0/46 (0%)	14/646 (2.1%)	0.6883

Statistically significant values (*p* < 0.05) are evidenced in bold

The mean (± SD) follow-up time was 46.1 (27.4) months, and at the time of follow-up 994 (77.5%) patients were dead. The mean (± SD) survival in the global cohort was 19.9 (± 22.4) months. Considering only dead patients, the mean (± SD) overall survival was 15.8 (± 18.8) months; a significantly greater survival was observed in females in comparison to males (19 ± 22.1 vs 21.8 ± 23 months; *p* = 0.003) and never smokers in comparison to ever smokers (26.4 ± 23.7 vs 21.1 ± 22.5 months; *p* = 0.003). Among patients with *EGFR* mutations, the eleven patients with exon 18 mutations had a significantly lower survival in comparison with those with exon 19 and exon 21 mutations (8.8 ± 5.7 vs 24.9 ± 20.9 months; *p* = 0.0023); no such difference was detected between the latter or between patients with *KRAS* exon 2 and exon 3 mutations. Finally, 193 patients were tested for the EGFR-T790M mutation at baseline—before the beginning of the systemic treatment and independently on the result of the *EGFR* testing for TKI-sensitizing mutations—by a NGS assay; among them, 26 (13.5%) had a very limited amount (very few mutated alleles) of the EGFR-T790M variant and showed a significantly lower survival in comparison to those without (8.7 vs. 47.4 months, *p* < 0.0001).

Kaplan–Meier global survival estimates in accordance with the genetic alterations detected are depicted in Table 3.

**Table 3:** Survival in accordance with the genetic alterations found in the study. Values are expressed in mean (confidence interval 95%) months

Gene	Altered	Wild type	*p*
*EGFR*	36.5 (30.6–42.4)	28.4 (25.5–31.4)	**< 0.0001**
*KRAS*	24.4 (19.4–29.4)	32.5 (29.0–36.0)	**0.0058**
*BRAF*	32.5 (17.3–47.7)	30.7 (27.6–33.8)	0.7337
*ALK*	20.4 (12.8–27.9)	25.6 (22.2–28.9)	0.6493
*MET*	23.4 (10.2–36.6)	25.9 (22.2–29.5)	0.8454
***EGFR***			
Males	33.9 (23.8–43.9)	26.4 (23.2–29.6)	0.0729
Females	36.7 (30.3–43.1)	31.9 (26.8–37.0)	**0.0017**
Smokers	33.1 (21.6–44.6)	26.4 (23.0–29.9)	0.1261
Never smokers	38.6 (30.3–46.8)	29.6 (20.6–38.6)	**0.0275**
***KRAS***			
Males	23.4 (18.7–28.1)	29.1 (25.2–33.1)	0.3026
Females	24.2 (12.7–35.6)	36.5 (31.4–41.7)	**0.0025**
Smokers	24.0 (18.8–29.1)	27.8 (23.8–31.8)	0.4366
Never smokers	13.1 (6.7–19.4)	34.7 (27.8–41.5)	**0.0055**

Statistically significant value is evidenced in bold

**Fig. 1:**
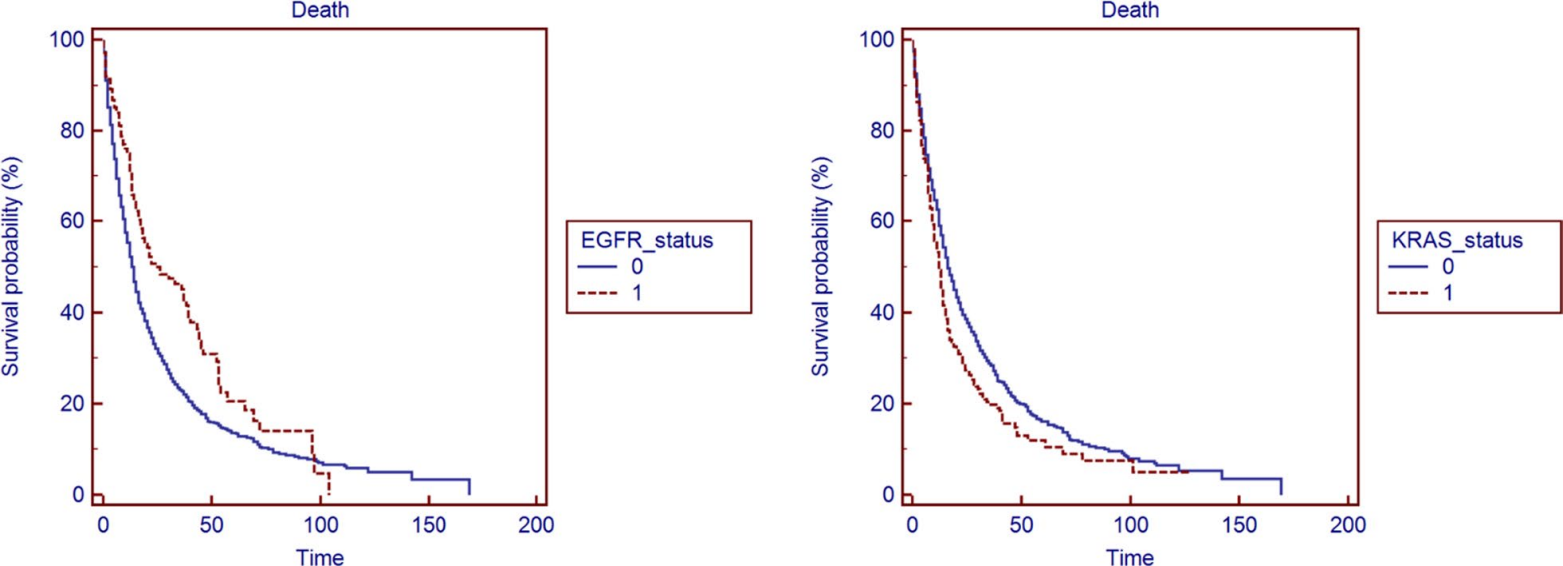
Kaplan–Meyer survival curves in patients with and without *EGFR* and *KRAS* mutations, respectively. Time is referred to follow-up months

**Fig. 2:**
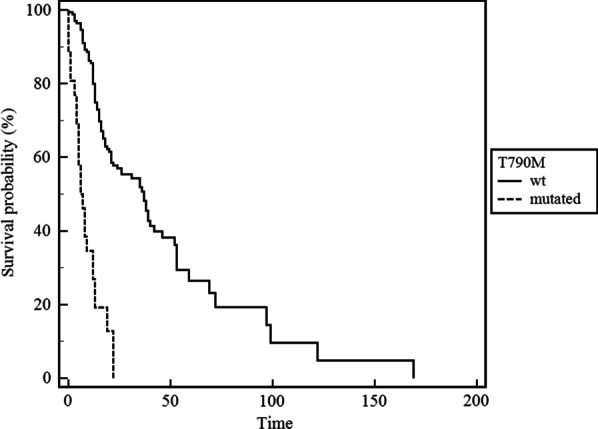
Kaplan–Meyer survival curves in patients with and without EGFR-T790M mutation. Time is referred to follow-up months

Kaplan–Meier estimates showed that patients harboring *EGFR* mutations had a significantly longer survival in comparison to those without (*p* < 0.0001), as opposed to *KRAS* mutations, which was associated with a significantly lower survival (*p* = 0.0058). The Kaplan–Meyer survival curves of *EGFR* and *KRAS* mutated patients in comparison to those with wild-type tumors are depicted in Fig. 1, while Fig. 2 depicts the survival curves of patients with EGFR-T790M mutation in comparison to those without. *EGFR* mutations provide globally a significant survival advantage in females and never smokers as opposed to *KRAS* mutations, which were shown to be negative prognostic factors in the same subsets. No statistically significant differences in survival were found regarding the other genetic alterations investigated. In the Cox regression model summarized in Table 4, survival rates were evaluated after adjustment for the genetic alterations under investigation as well as for age, sex and smoking status; in this model *EGFR* mutations represented the only parameter, which impacted significantly on global survival.

**Table 4:** Cox regression analysis including the main impacting factors and the genetic alterations under investigation

Factor	HR	95% CI	*p*
Age	0.9963	0.9846–1.0081	0.5352
Sex	1.1872	0.9033–1.5604	0.2184
Smoking status	1.0137	0.7085–1.4503	0.9407
*EGFR*	0.6254	0.4497–0.9561	**0.0283**
*KRAS*	0.9448	0.7419–1.2032	0.6453
*BRAF*	1.0292	0.5419–1.9546	0.9300
*ALK*	0.9213	0.5966–1.4227	0.7114
*MET*	0.8324	0.3904–1.7750	0.6349

Statistically significant value is evidenced in bold

## Discussion

Our study evidenced that, among the driver genetic alterations evaluated, in patients with lung adenocarcinoma the main factor that positively impacts real-life survival is the presence of an *EGFR* mutation in exons 18, 19 or 21, especially when associated with the absence of the T790M mutation in exon 20, no smoking history and female sex. This, in addition to the fact that *EGFR* mutations are the most common druggable genetic alterations in lung adenocarcinomas (10–16% in Caucasians, up to 78% in Asians [7, 8, 10]), and the fact that our series does not include patients treated with the third generation TKI Osimertinib, confirm that anti-EGFR treatments represent the cornerstone of modern targeted therapies in this setting. Other studies showed better survival rates in females and never smokers [11, 12].

In addition, in our series patients with tumors with exon *EGFR* 18 mutations had a worse survival in comparison to those with exon 19 and 21 mutations, as previously described [13]. Of note, in our study, 26 patients (13.5% of those tested) had tumors harboring the T790M mutation in the same tissue sample used for *EGFR* mutation analysis; despite a very few amount of mutated alleles being detected by NGS assay, such a subset of patients presented a consistently worse prognosis in comparison with cases who did not had the T790M variant. The EGFR-T790M mutation alone accounts for up to 50% of resistances in TKIs, which affect the majority of patients within the first year of treatment, and is considered quite rare at diagnosis, being the main mechanism of acquired (or secondary) drug resistance [14–16]. In this sense, our data show that, even if such a mutation is present at sub-clonal level in about one tenth of cases at the time of diagnosis, it may somehow affect the tumor behavior by causing intrinsic (or primary) drug resistance and inducing poor prognosis. This underlines the need of searching this mutation right from the initial molecular testing in order to both detect primary resistance to first and second generation TKIs and better classify the LAC patients from the prognostic point of view. Therefore, the occurrence of EGFR-T790M needs to be assessed despite the fact that last generation TKI drugs provide survival advantages in patients with early detected and persistent EGFR-T790M mutation [17]. Nevertheless, recent studies reported that the presence of EGFR-T790M in combination with complex *EGFR* mutations is a negative prognostic factor in patients treated with Osimertinib [18], suggesting that the need for EGFR-T790M testing will not come to an end with third generation TKIs. It appears, therefore, essential to use the biopsy or surgery samples to perform wider genetic testing that includes numerous *EGFR* variants and other genetic alterations which impact prognosis and survival, dependently or independently with the development of resistance to TKIs, like *ALK*, *KRAS*, *BRAF* and *MET* alterations [6]. The advent of next generation sequencing (NGS) technologies allows wider genetic evaluations, but it is currently expensive and not immediately available, especially in centers with low case numbers. The last version (4-2021) of the National Comprehensive Cancer Network recommends, when feasible, testing be performed via a broad panel-based approach, and, if not available, provides recommendations for other testing methodologies [5].

*KRAS* mutations have been traditionally considered a negative prognostic factor in patients with lung adenocarcinoma [7], currently representing the biggest challenge for the modern precision oncology. This is because *KRAS* mutations are generally mutually exclusive with *EGFR* mutations, as in our series, despite the results of some studies, which found a certain degree of co-occurrence [19, 20]. As a result, *KRAS* mutations generally affect consistent percentages of Caucasian (21–33%, 21.3% in our series) and to a lesser extend Asian (2–15%) patients with lung adenocarcinoma [7, 10], representing therefore an attractive target for future treatments. Several studies and a recent meta-analysis evidenced that *KRAS* mutations have a worse prognosis and a reduced or absent response to EGFR TKIs [21]. However, other studies failed to show a per se negative prognostic impact of *KRAS* mutations in patients with lung adenocarcinomas [22]. In our study, *KRAS* mutations were associated with worst prognosis, especially when associated with tobacco smoking, but they were not confirmed as independent negative prognostic factor of survival in Cox regression analysis.

Similarly, *BRAF* mutations and *MET* amplifications did not show an independent prognostic role in our cohort. Nevertheless, these alterations are consistently rarer in comparison to *KRAS* and *EGFR* mutations, and probably larger numbers are necessary to better evaluate their prognostic role. In our series, patients with *BRAF* and *MET* genetic alterations did not receive any targeted treatment because no such treatment were available at the time of the study, despite *BRAF* was a well-established therapeutic target in other malignancies like melanoma [23]. Currently, Dabrafenib and Trametinib are recommended for patients with a BRAF-V600E mutation, which was the only mutation detected in all the 28 cases (3% of the total tested) in our series [5]; this will surely improve the prognostic role of *BRAF* mutations in lung cancer in the future. *MET* amplifications were detected only in 14 cases (2% of those tested) in our series, a percentage consistently low considering that *MET* amplifications have been extensively documented as a mechanism of acquired resistance in 5–22% EGFR-mutated NSCLC upon therapeutic pressure with EGFR-TKIs; in addition, only two of these amplifications co-occurred with *EGFR* mutations, and thus, in patients subsequently treated with TKIs [24]. This suggests that, as opposed to the EGFR-T790M mutation, *MET* amplification is rarely present at the time of diagnosis and thus, it is a rare cause of primary resistance to TKIs. Currently drugs for MET exon 14 skipping mutations, which occur in 1–10% of the cases [24, 25], have been approved for clinical use (Capmatinib, Crizotinib, Tepotinib) [5], making these mutations and the study of acquired gene amplifications essential for the clinical management of the patients.

As opposed to *MET* alterations, *ALK* fusions represent a well-established therapeutic target in lung cancer, as they are detected in 3–7% of NSCLCs and have been associated with an absence of smoking, younger age, and adenocarcinoma histology [26]. In our study, *ALK* rearrangements occurred in 4.7% of the cases tested with no particular predilection for age, sex or smoking habits; two of the rearrangements occurred in concomitance with *MET* amplification. Curiously, the median survival of *ALK* wild type patients was higher, although not statistically significant, in comparison to those with *ALK* rearrangement. Actually, we do not have a full knowledge of the clinical history of the systemic treatment among such a subset of patients; therefore, we are not sure whether this unexpected lower survival among cases with *ALK* rearrangement might be somehow due to a real-life ineffectiveness of the first generation *ALK* inhibitors. Further real-life studies are necessary to better evaluate this finding, and to extend it to more recent second line medications.

Our study has some limitations, mainly its retrospective design and molecular testing based primarily on clinical needs, which limited the availability of samples for testing all the driver genes in all the cases. In addition, no liquid biopsies have been included in the study, and thus data on acquired EGFR-T790M—related resistance are not available. On the other hand, our study includes globally a relevant number of daily-practice cases, offering a unique insight on the impact of the main driver genetic alterations on the prognosis of lung adenocarcinoma.

## Conclusions

In conclusion, our results evidenced that the presence of *EGFR* mutations represent the most impacting, independent prognostic factor, regardless of the type of TKIs received and despite the occurrence of EGFR-T790M mutations in 13.5% of the cases tested—a percentage that appears higher than those reported in past studies though we classified positive cases using a very low threshold (at least 3 mutated alleles). Some of the genetic alterations (i.e. BRAF-V600 and KRAS-G12C) became recently druggable and this will certainly improve their role in improving overall survival. Data from future real-life studies based on a more comprehensive molecular classification at the time of diagnosis of the advanced disease are constantly necessary to better evaluate the real impact of specific genetic alterations and their corresponding targeted therapies in patients with lung cancer.

## Data Availability

All data generated or analysed during this study are included in this published article and its supplementary information files.
